# Study on Microwave-Assisted Ignition Using a Novel Aero-Engine Combustor

**DOI:** 10.3390/s23115056

**Published:** 2023-05-25

**Authors:** Yunwei Zhang, Bingbing Zhao, Liming He, Hao Zeng, Yipeng Chang

**Affiliations:** Aviation Engineering School, Air Force Engineering University, Xi’an 710038, China; zyw13889785364@buaa.edu.cn (Y.Z.); heliming369@163.com (L.H.); zwda693@163.com (H.Z.); kgdchangyipeng@163.com (Y.C.)

**Keywords:** microwave, plasma, ignition, combustor, resonator

## Abstract

Microwave plasma can improve the performance of ignition and combustion, as well as reduce pollutant emissions. By designing a novel microwave feeding device, the combustor can be used as a cavity resonator to generate microwave plasma and improve the performance of ignition and combustion. In order to feed the energy of microwave into the combustor as much as possible, and effectively adapt to the change in resonance frequency of combustor during ignition and combustion, the combustor was designed and manufactured by optimizing the size of slot antenna and setting the tuning screws, according to the simulation results of HFSS software (version: 2019 R 3). The relationship between the size, position of metal tip in the combustor and the discharge voltage was studied using HFSS software, as well as the interaction between ignition kernel, flame and microwave. The resonant characteristics of combustor and the discharge of microwave-assisted igniter were subsequently studied via experiments. The results show that the combustor as microwave cavity resonator has a wider resonance curve and can adapt to the change in resonance frequency during ignition and combustion. It is also indicated that microwave can enhance the discharge development of igniter and increase the discharge size. Based on this, the electric and magnetic field effects of microwave are decoupled.

## 1. Introduction

The combustor is a part of the core engine, and its performance directly determines the technical level of engine. Since the appearance of turbojet engine, the inlet temperature of combustor has increased from 450 K to above 900 K, and the outlet temperature has increased from 1100 K to above 2000 K. Higher combustor temperature requires better control in the intensity distribution of flame. At the same time, the inlet air velocity of combustor can reach 150 m/s, which is much higher than the flame propagation speed. In order to reliably ignite and stabilize flame, the air flow velocity has to be reduced by the diffuser and baffle plate, which leads to an increase in aerodynamic loss, thus affecting the efficiency of engine. At present, in order to meet the higher performance requirements, it is necessary to further improve the thrust weight ratio of engine and the performance for all working conditions. Therefore, this can only be achieved by reducing the size of combustor and realizing combustion regulation technology. However, it has to face the more severe challenges of unstable and uneven combustion. Moreover, in recent years, combustor ablation, blade fracture and other accidents have occurred frequently; therefore, a new ignition and combustion technology of aeroengine is urgently needed to obtain more uniform and effective combustion.

The traditional aeroengine always adopts the single-point ignition technology, namely spark ignition, which has the problems of unstable combustion, slow combustion and incomplete combustion under extreme operating conditions. The traditional solution is to improve the ignition energy, namely high-energy ignition, which leads to excessive energy consumption and greatly limits the performance of aeroengine. Recent studies on multi-point ignition show that it can reduce ignition delay time, broaden ignition boundary, reduce pollutant emissions, and improve combustion efficiency, which effectively solves the defects of single-point ignition. The traditional multi-point ignition is to increase the number of igniters; however, the effect is not ideal because the installation size on the engines is very limited.

Many researchers have summarized the basic principles of plasma ignition and assisted combustion [1,2,3,4,5,6]. Microwave plasma ignition and assisted combustion technology is a new technology developed following developments in the field of plasma in recent years. It can not only make the distribution of flame intensity uniform, accelerate propagation speed, improve combustion efficiency, broaden lean and rich burn flammability limits, reduce pollutant emissions, but also realize multi-point ignition and stable combustion. According to the classification of discharge devices, microwave plasma is mainly divided into three types, including microwave resonator torch, microwave radiation space and microwave-assisted plasma [7,8].

The researchers conducted experimental and numerical studies on different types of microwave resonator torch for ignition and assisted combustion [9,10,11]. The results show that the resonance frequency of the cavity will be reduced due to the formation of plasma; therefore, the input frequency will deviate from the resonance frequency, thus affecting the reflection coefficient and then reducing the output power of microwave. In the experiment, the resonance frequency decreases about 5 MHz after the formation of plasma. By controlling the input frequency to reduce the reflection coefficient, the energy input can be increased by 30%. Although microwave resonator torch can break down the air and ignite successfully, due to the limitation of the quality factor of cavity, high intensity of electric field and wide resonance bandwidth cannot coexist. Therefore, when the plasma forms and changes the resonance frequency of cavity, the requirements for ignition and assisted combustion cannot be met at the same time. Single-point ignition of microwave resonator torch can reduce ignition delay time, broaden burn flammability limits, accelerate combustion speed and stabilize flame, while multi-point ignition formed by microwave resonator torch can further enhance these advantages [12,13].

The research results [14,15,16,17,18,19,20] of microwave radiation space ignition and assisted combustion show that it shortens or even eliminates the laminar combustion stage, and directly enters the turbulent combustion stage, which can reduce fuel consumption by up to 30% and pollutant emissions by up to 80%. The combustion speed is much faster than that of ignition using a traditional spark plug, and the engine can be operated with extremely lean mixture [14]. The simulation results show that the intensity of electric field is related to the input microwave frequency and the height of cavity resonator. If they deviate, the intensity of electric field will decay rapidly [15]. Therefore, the shape design of cavity resonator for microwave radiation space is very important. For the combustor with fixed shape, the intensity of electric field in the cavity resonator cannot meet the requirements, which needs to change the design of microwave antenna to improve the intensity. Multi-point ignition of microwave radiation space can be also realized by setting several metal tips on the cavity resonator. Compared with the traditional ignition method, this method can shorten the time to reach the peak pressure by about 25% and increase the peak pressure by about 5% [16,17,18,19]. The research on microwave radiation space shows that it can accelerate the propagation speed of flame and make the flame larger and brighter, even if the low intensity of microwave electric field that cannot break down the air. When the high intensity can break down the air, it is able to keep the eddy current stable, which stabilizes the flame [20]. The research [20] also shows that plasma will greatly change the resonance frequency of cavity; therefore, it is important to adjust the resonance frequency of cavity.

The above research shows that microwave radiation space is the multi-point ignition, which can improve combustion efficiency, accelerate flame propagation, stabilize flame, and reduce pollutant emissions. However, the resonance frequency of cavity is related to the shape and working conditions, which has a great influence on the absorption of microwave energy. Therefore, what plays a crucial role in the practical application of microwave radiation space is to further study the relationship between resonance frequency and cavity shape, plasma parameters, so as to form the theory and method of assisted combustion under different working conditions.

The research on microwave-assisted plasma [21,22,23,24,25] shows that microwave-assisted ignition can successfully ignite with less ignition energy, and increase the volume and lifetime of plasma at high pressure, so as to obtain faster, more complete and stable combustion, and have the ability of ignition at high pressure [21]; however, the influence of microwave will gradually decrease with the increase in pressure [22,23]. Meng Y [24,25] has studied microwave-assisted combustion via plasma igniter. The results show that microwave accelerates the combustion speed and uniformities the distribution of flame intensity, the combination of microwave and plasma igniter can reduce power consumption.

Microwave resonator torch is a single-point ignition with small ignition area. Although microwave radiation space ignition is a multi-point ignition method, it is difficult to form the high intensity of electric field because of the large area of combustor, so that only argon and other gases easy to be broken down can be used for ignition. The advantage of microwave radiation space is that it can form a large area of microwave electric field for assisted combustion. Microwave-assisted plasma can reduce the power consumption of igniter, improve the ignition performance, and also play a good role in assisted combustion, which can be realized by radiating the igniter with the microwave antenna directly, or by the resonant electric field in combustor. Within relevant research, radiating the igniter with the microwave antenna directly has been explored, only a single-point ignition has been formed, and it has great influence on flame and aerodynamic, because it is too close to the igniter. By comparison, microwave-assisted plasma via microwave radiation space can form the resonant electric field in the combustor, which has a relatively high intensity and can form multi-point ignition, and will not affect the flame and aerodynamic. Therefore, it is a very potential technology for ignition and assisted combustion.

## 2. Design Considerations and Description for the Combustor as Microwave Cavity Resonator

An X-slot antenna is invented. Compared with a single slot antenna, the X-slot antenna can form a more powerful resonant electric field and has a better antenna directivity. Its size matches with that of combustor, which can form the high intensity and large area of electric field and magnetic field in the combustor for assisted ignition and combustion. If several metal tips are set on the combustor, the electric field of tip formed via microwave radiation is enough to break down the air for discharge, and can form multi-point ignition. The combination of the X-slot antenna and combustor can become a novel combustor and quickly enter the practical application stage.

**Figure 1:**
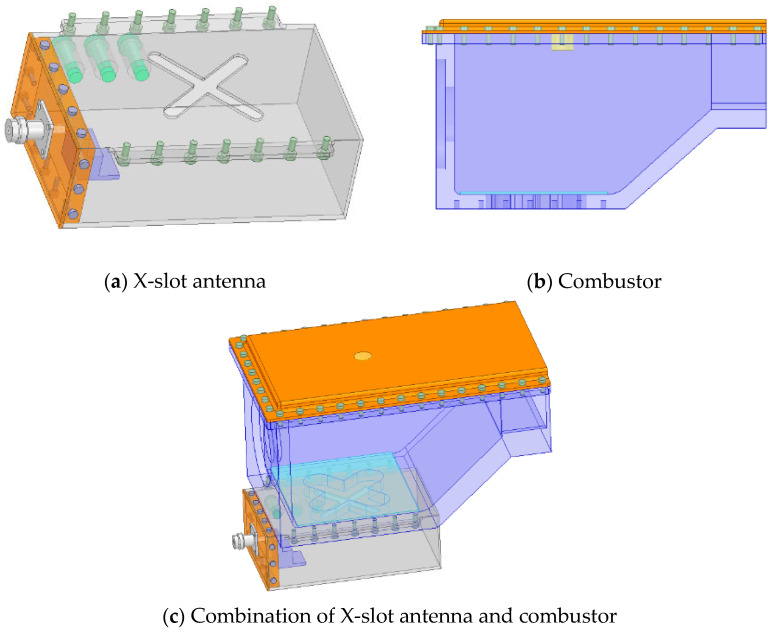
Structure of microwave antenna and combustor.

As shown in Figure 1, the waveguide is connected to 2.45 GHz microwave power supply through the coaxial line with TNC connector. A microwave is fed into the combustor through the X-slot. At the same time, the quartz glass is used to isolate the gas and X-slot, so that the microwave can penetrate the quartz glass into the combustor, and will not cause gas leakage. The size of X-slot on the combustor is larger than that of the waveguide, which is designed to further improve the resonance. The three tuning screws on the side of waveguide can adjust the resonance frequency of combustor to adapt the changes under different working conditions. The igniter is installed at the top of combustor, facing the X-slot below. Because of the special size of combustor and relative position between the X-slot and igniter, a non-standard waveguide is designed to make microwave resonate.

The combustor with large volume has a lower quality factor. By designing the microwave antenna reasonably, the combustor can obtain a wider resonance curve, which can deal with the influence of resonance frequency drift caused by plasma, thus avoiding the total reflection of microwave energy, thus feeding most or even all of the microwave energy into the combustor, which is conducive to microwave-assisted ignition and combustion. The way to achieve resonance with a wider resonance curve is different from that to change the input frequency with a control device [9]. The method carried out with a wider resonance curve has a simpler structure and more reliable performance. It can not only reduce control elements, but also have a wider operating frequency range after adding tuning devices, which is more suitable for the requirements of microwave-assisted ignition and combustion. Therefore, the design principle of combustor as microwave cavity resonator in this paper is to obtain a wider resonance curve and the higher intensity of spatial electric field. Because two slots can feed the microwave energy, the intensity and directivity of electric field are better than those of single slot. The X-slot is placed on the middle of combustor, which can have not only the high intensity of resonant electric field, but also a good position to radiate the igniter. It is a good choice for microwave-assisted ignition and combustion.

## 3. Simulation Analysis

HFSS (high frequency structure simulator) is a widely used electromagnetic simulation software that meets industrial standards for three-dimensional electromagnetic field design and analysis. The electromagnetic performance of products designed using this software is similar to the simulation results. Through simulation calculations, various performance parameters of the cavity resonator can be obtained, such as gain, reflection coefficient, image of electric field vector, image of magnetic field vector, standing-wave ratio, impedance, resonance frequency, directionality, and bandwidth. The simulation for the electromagnetic field is based on the solution of Maxwell’s equations and consists of the following four equations:

(1) $$\nabla \times H(r,t)=\frac{\partial }{\partial t}D(r,t)+J(r,t)$$

(2) $$\nabla \times E(r,t)=-\frac{\partial }{\partial t}B(r,t)$$

(3) ∇×D(r,t)=ρ(r,t)

(4) ∇×B(r,t)=0

where *E* (*r*, *t*) is the electric field intensity, *B* (*r*, *t*) is the magnetic flux density, *H* (*r*, *t*) is the magnetic field density, *D* (*r*, *t*) is the electric displacement, and *J* (*r*, *t*) is the current density, *ρ* (*r*, *t*) is the charge density, *r* and *t* represent the position and time, respectively.

The calculation process of HFSS software is as follows:

(1) Set the type of solution.
(2) Create a simulation model, mainly including drawing the geometric shape of the model, setting the location and type of excitation ports, boundary conditions, and materials.
(3) Solution settings, mainly including setting the minimum number of iterations, convergence accuracy, solution frequency, geometric parameter scanning and optimization, etc.
(4) Simulation calculation.
(5) Data post-processing, mainly including gain, reflection coefficient, image of electric field vector, image of magnetic field vector, standing-wave ratio, impedance, resonance frequency, directionality, and bandwidth.

The simulation model in this article mainly studies the electromagnetic characteristics inside the cavity resonator. However, due to the connection between the exhaust ports of the combustor and the free space outside, the radiation boundary condition is selected for the simulation model to consider the impact of the far-field region. The material of combustor is metal, and its surface is automatically defined as the boundary condition named finite conductivity. At the same time, causal material settings are applied. The conductivity, relative dielectric constant and other parameters of the transmission medium and metal surface change with the frequencies, which is more consistent with a real situation of electromagnetic wave propagation and loss.

After optimization using HFSS software, the size, position of X-slot and waveguide are optimized when combined with the combustor, and the reflection coefficient curve is obtained, in addition to the resonance frequency range that can be tuned by screws. The images of electric field vector and magnetic field vector are also obtained, as well as their intensities.

### 3.1. Performance of Combustor as Microwave Cavity Resonator

*S*(1,1) is the reflection coefficient, which is calculated with the following equation:

(5) $$S(1,1)=10\mathrm{lg}\frac{P_{r}}{P_{i}}$$

where *P*_*i*_ is the output power of the emitting port of microwave, *P*_*r*_ is the reflected power back into the emitting port of microwave.

**Figure 2:**
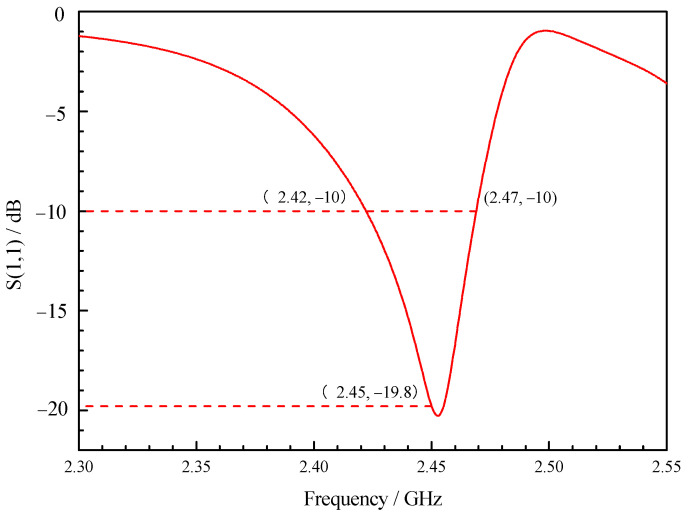
Reflection coefficient curve of the combustor with cavity.

**Figure 3:**
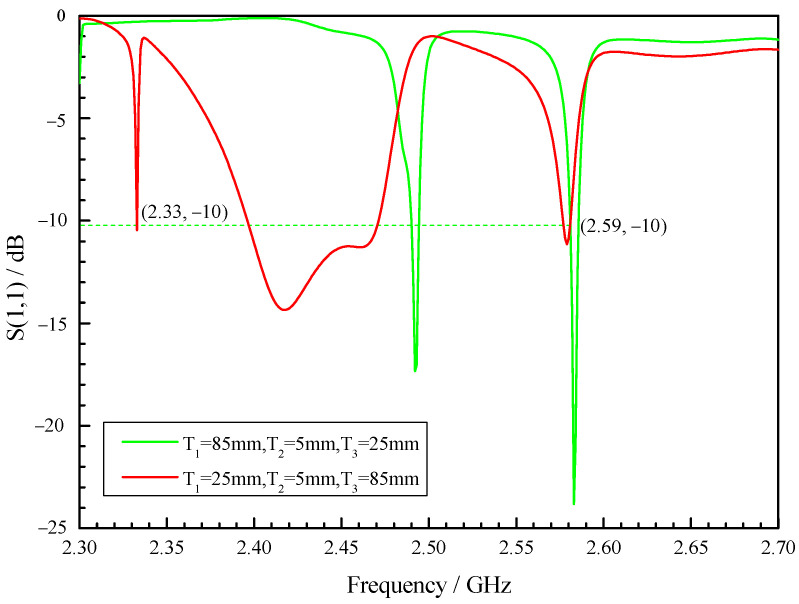
Reflection coefficient curve of the combustor with cavity by tuning (T_1_, T_2_, T_3_ are the length of screws into the combustor).

As shown in Figure 2, the simulation results show that the reflection coefficient of the optimized X-slot antenna is less than −10 dB when the operating frequency is within the range of 2.42 GHz to 2.47 GHz, indicating that the antenna feeds more than 90% of the energy into the combustor. Therefore, the antenna has the operating bandwidth of 50 MHz without the use of tuning screws, which can meet the change in resonance frequency under different working conditions. As shown in Figure 3, the tuning screws can greatly broaden the bandwidth of X-slot antenna so that its reflection coefficient is less than −10 dB when the operating frequency is between 2.33 GHz to 2.59 GHz, which means the operating bandwidth reaches 300 MHz. Therefore, it becomes an ultra-long bandwidth antenna which can be used to assisted ignition and combustion effectively. It is very meaningful for practical applications.

**Figure 4:**
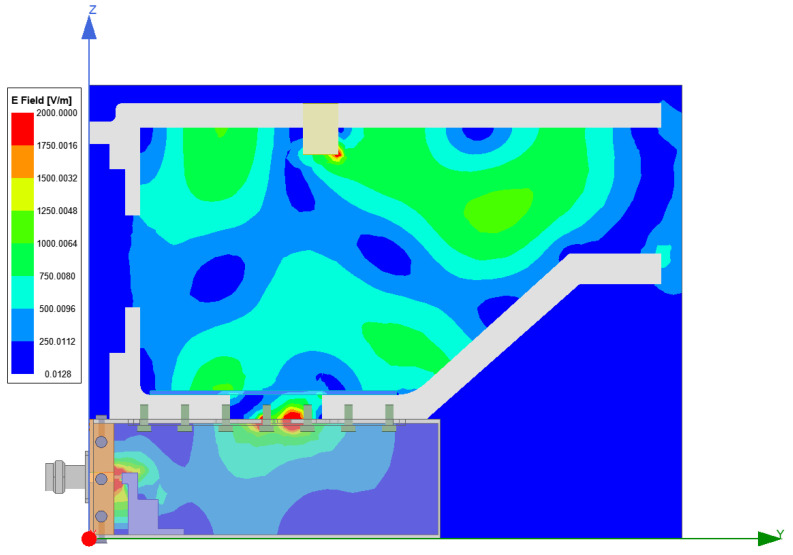
Distribution of the electric field intensity inside the combustor with cavity.

**Figure 5:**
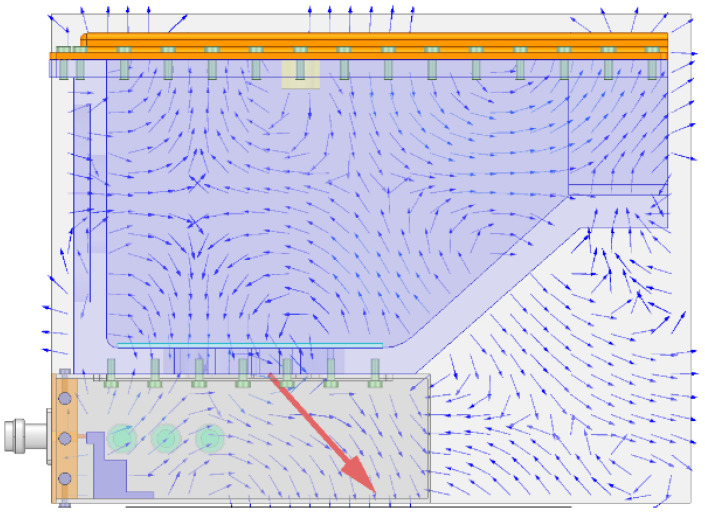
Electric field vector inside the combustor with cavity.

The distribution of the electric field in the combustor is studied via simulation. As shown in Figure 4, when the microwave power supply is 1 W, the intensity of electric field in the combustor is at most about 1200 V/m, and is widely dispersed in the combustor, which can well meet the requirements of assisted combustion. The indicator is at the forefront of similar research. As shown in Figure 5, the image of electric field vector shows that the microwave has formed several electric field vortices in the combustor, which are similar to the flow vortices in shape. In the case of low fluidity, the role of magnetic field can be ignored; therefore, the Coulomb force generated by the electric field will dominate the formation of vortices. From the view of Reynolds number, the influence of Coulomb force is equivalent to the increase in inertial force, which increases the Reynolds number and further forms turbulence. Therefore, when the microwave power and the intensity of electric field increase, the turbulence intensity will increase and a stronger turbulence will be formed [26].

**Figure 6:**
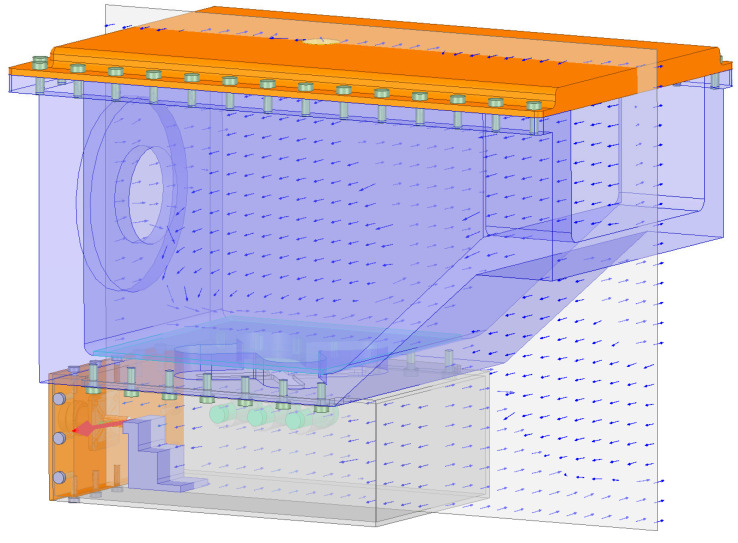
Magnetic field vector inside the combustor with cavity.

As shown in Figure 6, the image of magnetic field vector shows that the microwave not only forms the eddy current of electric field in the combustor, but also forms the magnetic field. From Figure 6, we can see that the direction of magnetic field is always perpendicular to that of the flow direction. Therefore, when the fuel has strong fluidity, the direction of Lorentz force is always perpendicular to that of the flow direction; thus, the electrons and free radicals generated by discharge will flow under the influence of Lorentz force. Therefore, the interaction between particles improves the stability of eddy currents. From the view of Reynolds number, the influence of Lorentz force is equivalent to increasing the viscous force, thus reducing the Reynolds number and preventing turbulence separation. The increase in viscous force is realized by the collision between free electrons, radicals and other molecules. The value of viscous force has a great relationship with the flow speed at which the particles cut the magnetic field. The above analysis can reveal the mechanism of enhancing turbulent combustion via microwave [14] and the mechanism of stabilizing flowing flame via microwave discharge [20].

### 3.2. Metal Tip Discharge

**Figure 7:**
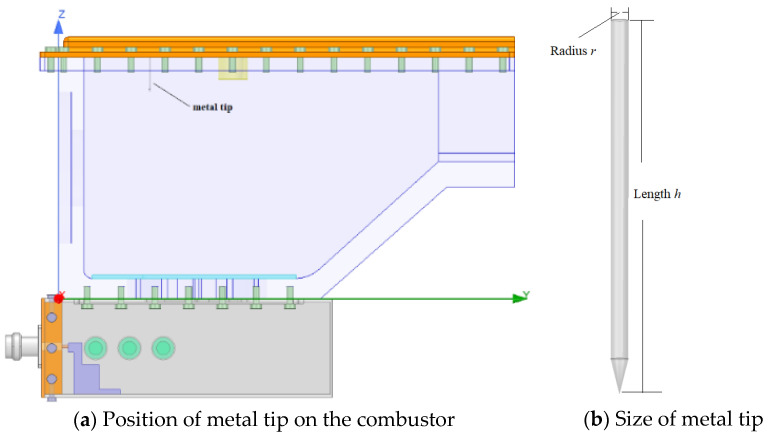
Metal tip design inside the combustor with cavity.

Only under the condition of discharge, can the microwave stabilize the flame [20]. Therefore, whether from the consideration of multi-point ignition or assisted combustion, the combustor should have several points to discharge, and the discharge points can be increased by setting several metal tips on the combustor, as shown in Figure 7.

Sharp tips are easier to discharge [27], and the antenna with a quarter of wavelength of electromagnetic wave has the highest efficiency in receiving and converting, according to the transmission line theory. Therefore, the discharge performance of metal tip depends on the taper of tip and the length of metal. By designing the size and position of the metal tip reasonably, discharge can be carried out in the combustor. The intensity of electric field with different size and position is simulated using HFSS software. The results show that the intensity can reach that of air breakdown under atmospheric pressure. Therefore, the combustor with metal tips is equivalent to the combination of microwave radiation space and microwave resonator torch, which has both advantages and avoids both disadvantages.

**Figure 8:**
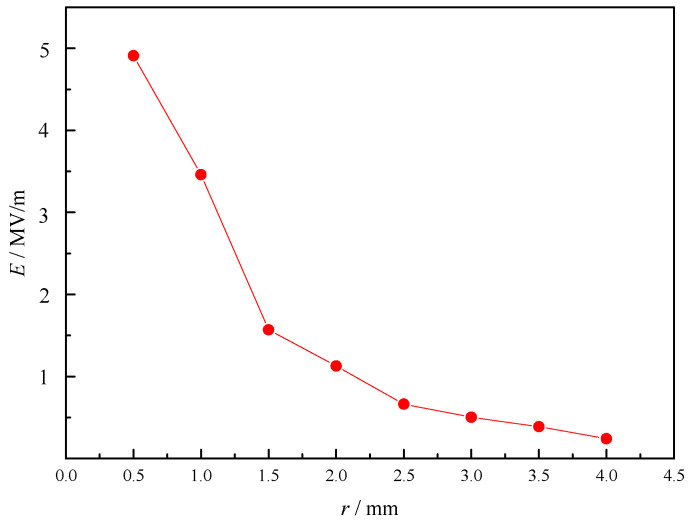
Intensity of metal tip discharge changes with radius.

**Figure 9:**
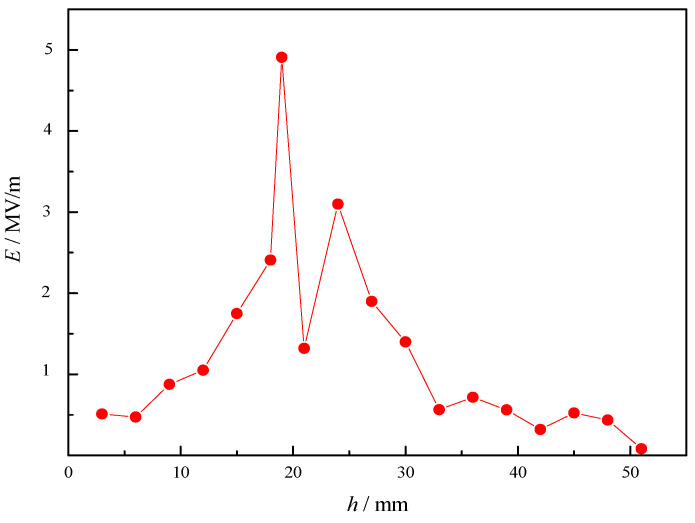
Intensity of metal tip discharge changes with length.

**Figure 10:**
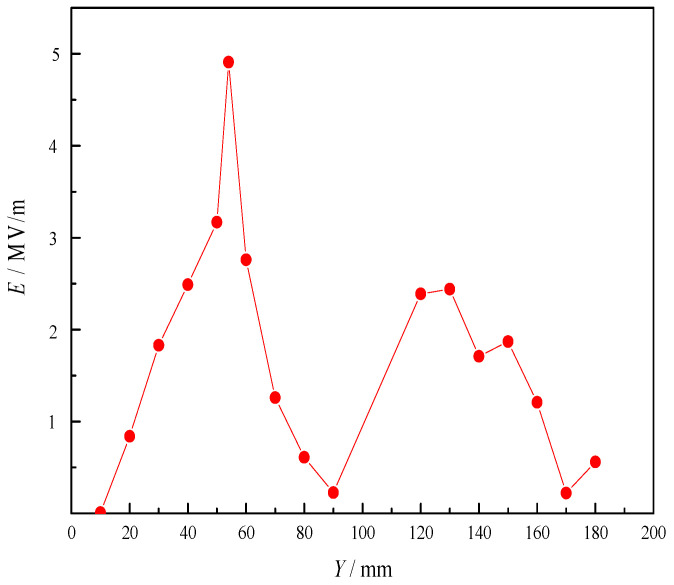
Intensity of metal tip discharge changes with distance.

As shown in Figure 8, the intensity of electric field (*E*) of metal tips increases with the decrease in radius (*r*) of metal cylinder, which means that the increase in taper can improve the intensity. As shown in Figure 9, the intensity also changes with the length (*h*) of cylinder. When its length reaches a certain value, the intensity is the highest. As shown in Figure 10, the intensity at different horizontal positions *Y* is also different. This is because the electromagnetic field of microwave in the combustor is uneven, and the intensity is higher at the position where the electromagnetic field of microwave is higher.

In the research of multi-point ignition in cylinder combustor, the experimental results show that multi-point ignition can improve the combustion speed and efficiency, while microwave discharge can stabilize the flame. Therefore, the method of setting several metal tips is particularly suitable for igniting high-speed flow in aeroengine combustor, which can effectively improve the burn flammability limits and flame stability. From the results of design analysis of metal tips, we can see that its size is much smaller than that of the igniter in the combustor of aeroengine, and its influence on the airflow will be relatively small. At the same time, its mass increase will also be smaller. Compared with the traditional multi-point ignition, it has many advantages. If a large number of settings are made, the combustion efficiency and stability can be improved, and then the thrust weight ratio can be improved. Only discharge can stabilize the flame; however, if the microwave energy is widely dispersed in the combustor, it is difficult to form the intensity of electric field to break down the air; nevertheless, metal tips’ discharge can reach the intensity, meaning it can play a role in stabilizing the flow field. The increase in combustion speed and the stability of flame can reduce or even replace the turbulator, such as V-cone or cavity, which is helpful for forming a more compact, simple and light combustor.

### 3.3. Interaction between Microwave and Plasma

**Figure 11:**
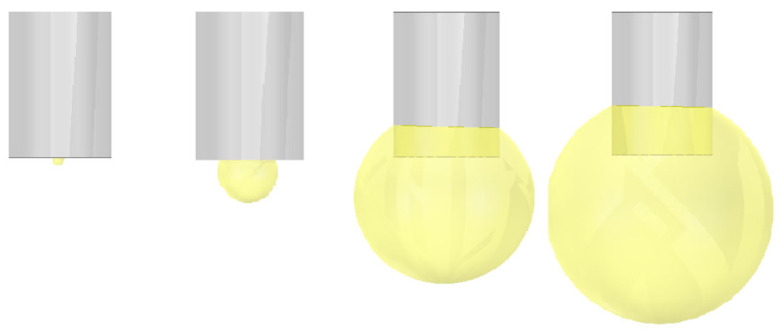
Physical model of the ignition kernel.

The process of ignition is that the igniter breaks down the air to form a plasma discharge, and the radius of discharge kernel increases sharply, igniting the surrounding gas to form an ignition kernel. When the radius of ignition kernel reaches the minimum ignition radius, the fuel will be ignited to form a flame. As shown in Figure 11, a simplified discharge model of spherical flame is established according to the formation law of discharge kernel. The simplified model of discharge is that the electron density and temperature remain constant and will not change with the enlargement of the volume. According to the spark ignition parameters described in ref. [28], this value is set as *T*_e_ = 3 eV *N*_e_ = 4 × 10^21^ m^−3^. These parameters will determine the conductivity and relative dielectric constant of the discharge model.

(6) $$\upsilon_{e}=\sqrt{\frac{8}{km_{e}\pi }}sp\frac{\sqrt{T_{e}}}{T}$$

where *υ*_*e*_ is the collision frequency, *k* is the ideal gas constant, *s* is the area of effective collision cross section between electron and neutral particle, *p* is the gas pressure, *T* is the gas temperature, and *T*_*e*_ is the electron temperature.

(7) $$\sigma =\frac{e^{2}n_{e}\upsilon_{e}}{m_{e}\left(\omega^{2}+\upsilon_{e}{}^{2}\right)}$$

where *σ* is the conductivity, *ω* is the angular frequency of microwave, *e* is the electron charge, *n_e_* is the electron density, *m*_e_ is the electron mass.

(8) $$\varepsilon_{r}=1-\frac{e^{2}n_{e}}{m_{e}\varepsilon_{0}\left(\omega^{2}+\upsilon_{e}{}^{2}\right)}$$

where *ε_r_* is the relative dielectric constant.

**Figure 12:**
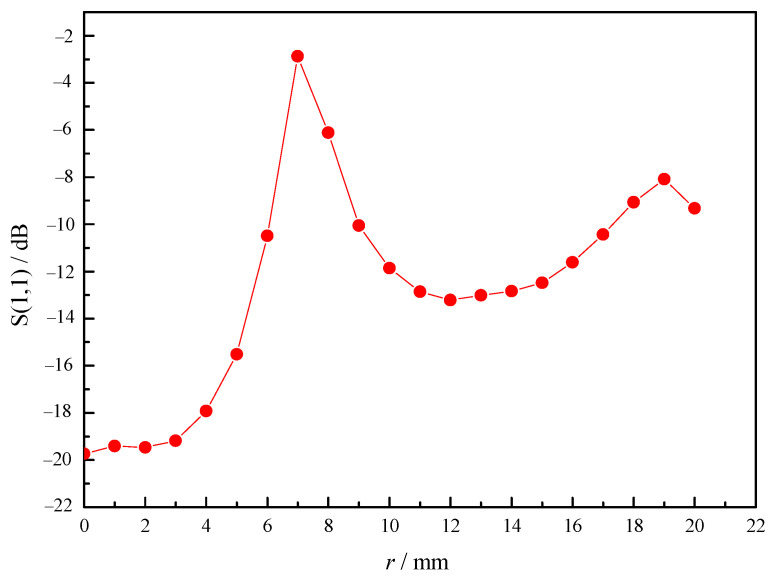
Reflection coefficient changes with the radius of ignition kernel.

Taking the electron density and temperature of the discharge model into the above formula, the conductivity and relative dielectric constant of the model can be estimated and set in the material parameters of HFSS software. The reflection coefficient of microwave for different discharge kernel radius will be obtained, and the influence of the development of discharge kernel on the microwave propagation can be studied. As shown in Figure 12, the simulation results show that with the increase in discharge kernel radius, the reflection coefficient decreases sharply, and then increases with the increase in the radius; however, it cannot return to the initial state. The reason is that the plasma with high electron density has a great influence on the phase of microwave propagation [29], which leads to the failure of resonance between the incident wave and the reflected wave, thus increasing the reflection coefficient. With the increase in discharge kernel volume, the phase changes and the reflection coefficient increases gradually. When the phase changes to a certain extent, the combustor enters the resonance state again. However, because the shape of discharge kernel, there are different effects on the phase of microwave propagation at different positions, and the resonance cannot become the initial state. At this time, the existence of tuning screws can play a role in adjusting the reflection coefficient. This is consistent with the experimental research in ref. [11,20,30].

**Figure 13:**
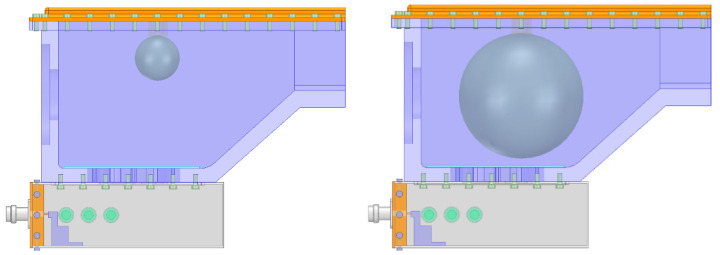
Physical model of the flame.

**Figure 14:**
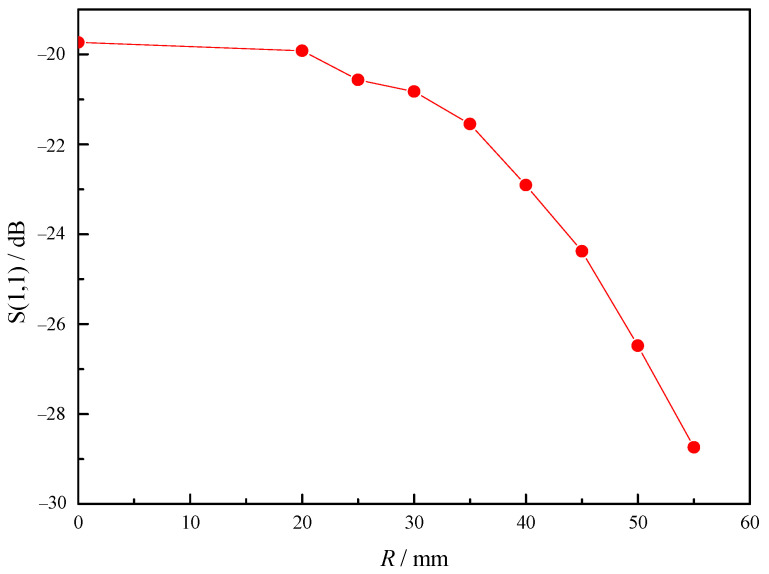
Reflection coefficient changes with the radius of flame core.

The same method was adopted to study the influence of the development of a spherical flame on microwave propagation, as shown in Figure 13. The physical model of a spherical flame was established. The study in ref. [31] shows that the flame is a non-equilibrium plasma with low electron density. They are measured as follows: *T_e_* = 1.3 eV, *N_e_* = 5 × 10^16^ m^−3^. As shown in Figure 14, the simulation results show that the reflection coefficient decreases with the increase in flame radius. The reason is that the plasma with low electron density has little influence on the phase of microwave propagation, especially when the collision frequency is high [29]. The flame just meets the two conditions of low electron density and high collision frequency; therefore, it will not affect the resonance of the combustor. However, the electron density of the flame is higher than that of air, which increases the microwave absorption; therefore, the reflection coefficient decreases with the increase in flame volume. This is consistent with the experimental research in ref. [20].

The simulation results also show that the resonance frequency of combustor will not change with the development of flame, but will change with the development of discharge kernel during ignition. When the radius of discharge kernel is 4 mm, it will decrease by about 4 MHz, which is consistent with the research in ref. [9]. The research on spark discharge in ref. [32,33] shows that the radius of discharge kernel does not exceed 4 mm even for microwave-assisted discharge. Therefore, the bandwidth of combustor as microwave cavity resonator is far wider than the range of resonance frequency, which can ensure the efficient feeding of microwave energy, which solves the problem for application of continuous microwave. If pulse microwave is used, it will also play a better role. For plasma discharge ignition, which is carried out with a larger discharge kernel and higher electron density, the change in resonance frequency will be out of the bandwidth; however, it still does not exceed the bandwidth after tuning the screws. The combustor as microwave cavity resonator can be used for equilibrium and non-equilibrium plasma ignition, and has very high application value.

The coupling of microwave and plasma is a very complex problem. Microwave will promote the generation of plasma in the process of propagation; therefore, it changes the conductivity and relative dielectric coefficient of plasma, which in turn affects the propagation of microwave. The increase in microwave power and resonance frequency will enhance the plasma discharge; therefore, the angular frequency of plasma determined by electron density becomes an important factor in the coupling of plasma and microwave energy with the increase in discharge. The increase in angular frequency caused by higher electron density can make more microwave energy be absorbed. However, for microwave cavity resonator, with the generation of plasma, the resonance frequency will drift due to the scattering effect, and the microwave energy will then be almost totally reflected due to the deviation from resonance frequency, which will lead to less microwave energy being absorbed. Therefore, the pulsed microwave power supply can generate plasma better when plasma is coupled with microwave, because it can repeatedly generate plasma without total reflection. There is an optimal pulse width according to the settings of power, duty cycle and pulse period; therefore, most of the microwave energy can be fed into the discharge or ignition kernel by controlling the pulse width base on the plasma cooling time [34,35].

## 4. Experimental Test

### 4.1. Resonant Characteristics of the Combustor as a Cavity Resonator

**Figure 15:**
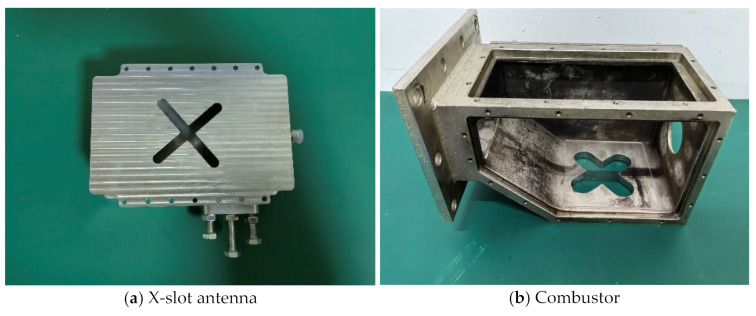

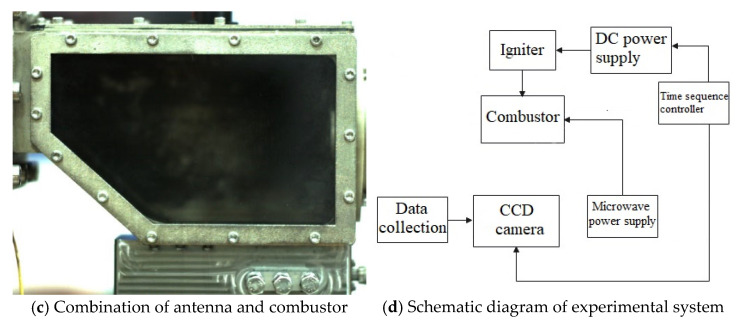
Experimental device and schematic diagram of experimental system.

In order to verify whether the performance of the combustor as microwave cavity resonator reaches the design indicators, the X-slot antenna matching the combustor was produced according to the simulation size and the experiment was conducted, as shown in Figure 15. The waveguide is made of aluminum, the X-slot is set on the center, and three tuning screws are set on the side. The X-slot antenna is located under the bottom of the combustor and connected by screws. The X-slot is isolated from the combustor via JGS1 quartz glass, and the two sides of combustor and the top wall panel are designed to be removable. The right side is the inlet of gas, and the left side is the outlet. The top wall panel can place an igniter. The design value of resonance frequency is 2.45 GHz.

**Figure 16:**
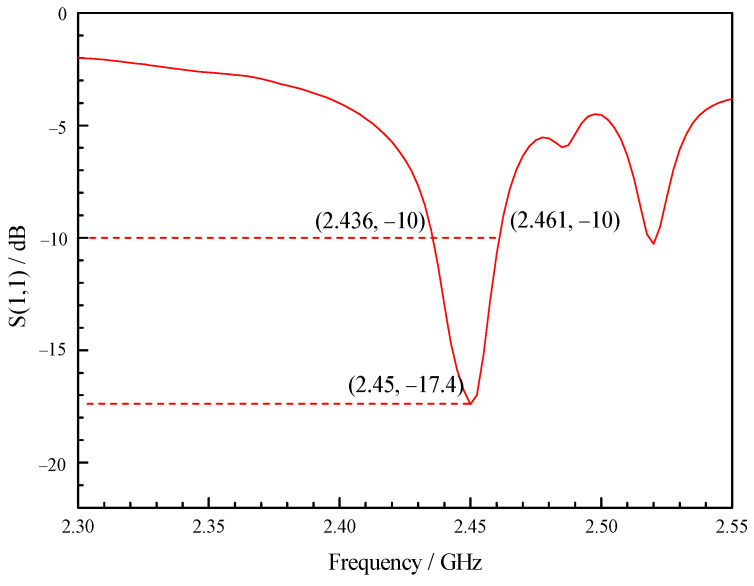
Reflection coefficient curve of the combustor with cavity in the experiment.

**Figure 17:**
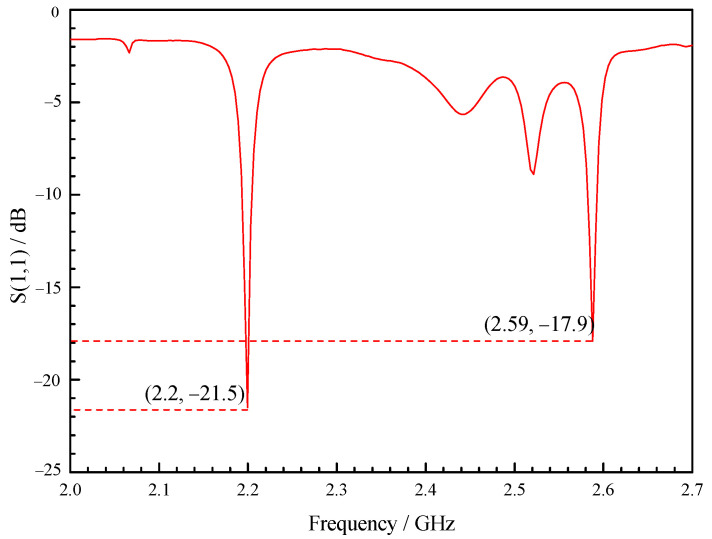
Reflection coefficient curve of the combustor with cavity by tuning in the experiment.

The experimental research was carried out using Anritsu MS46122B vector network analyzer. The resonance frequency, bandwidth and tunable range of combustor were measured, and the testing error of vector network analyzer is ±0.5 dB. As shown in Figure 16, the experimental results show that due to the accuracy of manufacturing, the resonance frequency of the combustor is slightly offset; however, the combustor can be resonant at 2.45 GHz by slightly tuning the rightmost screw, and the bandwidth is 25 MHz, ranging from 2.436 GHz to 2.461 GHz. As shown in Figure 17, when only the rightmost screw is used for tuning, the tuning range can be from 2.2 GHz to 2.59 GHz. At present, for most of the relevant researchers, it is difficult to make the combustor resonate from ignition to combustion all the time. This wide bandwidth combustor can make the combustor in the resonant state regardless of whether plasma is generated, which can effectively improve the influence of microwave on ignition and combustion. The design of a double slot can improve the intensity of regional resonant electric field, and the increase in intensity of reduced electric field can play a role in improving the non-thermal effects of plasma [36,37,38,39]. Higher intensity can also increase the peak power, and the increase in peak power is conducive to the absorption of microwave energy by plasma [40]. The ultra-long tuning range brought on by the tuning screws makes it suitable for various working conditions, including discharge, ignition and combustion. Tunable combustor as microwave cavity resonator is a novel combustor with great potential.

The bandwidth of combustor as microwave cavity resonator obtained from simulation is 2.42~2.47 GHz, as shown in Figure 2. The bandwidth obtained from the experimental research is 2.436~2.461 GHz, as shown in Figure 16. Therefore, the relative error of bandwidth obtained from simulation is −6.6~3.7%.

### 4.2. Microwave Assisted the Discharge of Ignitor

The relevant research on microwave-assisted combustion shows that the effect of microwave only occurs in the early stage. In order to study the mechanism of microwave-assisted ignition and combustion, the spark discharge of microwave-assisted high-energy igniter was studied using a high-speed camera. The experiment is carried out with the combustor of Figure 15. The high-energy igniter generates spark discharge through the DC power supply, and the microwave antenna is powered by the microwave power supply (power 0~200 W, duty ratio 0~100%, pulse frequency 1~3000 Hz) to transmit microwave for radiating the igniter. The high-speed camera obtains the image of spark discharge. The start time and end time of whole system are controlled through the timing controller.

**Figure 18:**
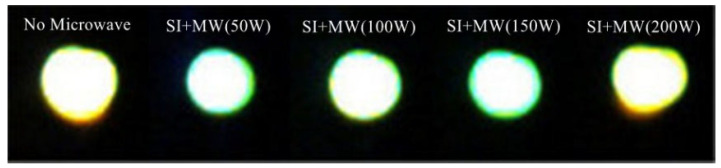
The image of spark discharge kernel with microwave-assisted.

**Figure 19:**
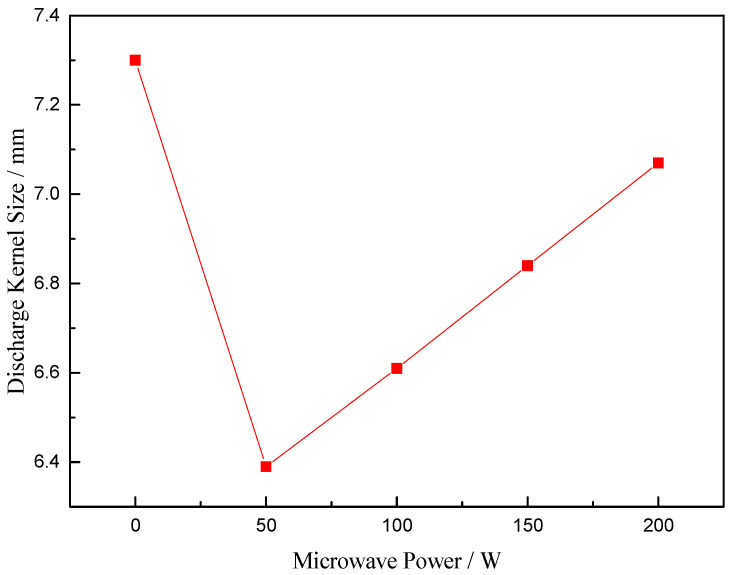
The size of spark discharge kernel with microwave-assisted.

Figure 18 shows the image of discharge kernel without microwave-assisted and with microwave; the power is 50 W, 100 W, 150 W and 200 W, respectively. The images in Figure 18 are those with maximum discharge size for each condition to represent the maximum ignition capacity of igniter. The size of discharge kernel will be measured via the maximum horizontal size, and the image in Figure 19 is the size value of discharge kernel dealt with image-pro software. We can see that when the power supply of the high-energy igniter is unchanged, the size of discharge kernel assisted by microwave decreases; however, with the increase in microwave power, the size gradually increases.

This phenomenon indicates that microwave can enhance the development of spark discharge, thus reducing the size of discharge kernel. However, when the microwave energy is further increased, stronger ionization will lead to the increase in discharge kernel size. Microwave establishes an electric field and a magnetic field in the combustor. Because the energy coupling between microwave and spark discharge is mainly through electric field for energy exchange, the increasing size is attributed to the microwave electric field, while the decreased size is attributed to the magnetic field because it can enhance the development of spark discharge. Because the effect between high frequency magnetic field and ions can be ignored, according to the analysis using the Townsend discharge model, the mechanism between magnetic field and electrons may be that the magnetic field changes the ionization path of electrons, resulting in more collision ionization in unit space, thus enhancing the development of spark discharge, which will lead to the increase in discharge power density and a wider ignition limit. The increase in discharge power density will cause more complete and rapid combustion. At the same time, the discharge kernel with microwave-assisted is smoother; however, with the increase in microwave power, the wrinkles of discharge kernel increase. We believe that this is caused by the two-stream instability of plasma. When the relative motion speed between electrons and ions is large, the plasma vibration will occur. We call this the two-stream instability of plasma, which is an important mechanism of turbulent heating. The turbulent heating will cause the turbulent combustion. Microwave causes more collisions and ionization in unit space; therefore, the relative motion speed between electrons and ions decreases, and plasma vibration decreases. With the increase in microwave power, stronger ionization causes the relative motion speed between electrons and ions to increase, and plasma vibration increases.

The formula for calculating the size of discharge kernel is:

(9) $$S=\frac{W}{Pix_{1}}Pix_{2}$$

where *S* is the size of discharge kernel, *W* is the width of combustor measured using a steel ruler, *Pix*_1_ is the pixel value of the width of combustor, and *Pix*_2_ is the pixel value of the horizontal size of discharge kernel. Therefore, the relative error in calculating the size of discharge kernel is:

(10) $$\frac{\Delta S}{S}=\sqrt{{\left(\frac{\partial S}{\partial W}\right)}^{2}{\left(\frac{\Delta W}{S}\right)}^{2}+{\left(\frac{\partial S}{\partial Pix_{1}}\right)}^{2}{\left(\frac{\Delta Pix_{1}}{S}\right)}^{2}+{\left(\frac{\partial S}{\partial Pix_{2}}\right)}^{2}{\left(\frac{\Delta Pix_{2}}{S}\right)}^{2}}$$

The measurement error (Δ*W*) of the width of combustor measured using the steel ruler is 0.5 mm, and the reading error of pixel value (Δ*Pix*_1_ and Δ*Pix*_2_) is 0.5; therefore, the relative errors of the discharge kernel size without microwave-assisted and at microwave power 50 W, 100 W, 150 W, and 200 W are calculated to be 1.61%, 1.83%, 1.77%, 1.71%, and 1.66%, respectively.

## 5. Conclusions

In this paper, numerical simulation is used to study the design principle of combustor as microwave cavity resonator, electric field, magnetic field, performance of metal tip discharge, and interaction between microwave and plasma. The verification is also carried out via experiments. The conclusions are as follows:

(1) The combination of X-slot microwave antenna and combustor has a large bandwidth, which can adapt the changes in resonance frequency caused by plasma and form the high intensity of electric field in the combustor. The setting of tuning screws can greatly widen the resonance frequency range of combustor.
(2) Under the action of microwave, a several eddy current of the electric field similar to that of flow is formed in the combustor, and the magnetic field perpendicular to the direction of gas flow is also formed. Charged particles will be affected by the corresponding Coulomb force and Lorentz force, which can effectively explain the phenomenon that microwave can accelerate the formation of turbulent combustion and stabilize flame.
(3) The discharge performance of the metal tip depends on the taper of tip and the length of the metal. By designing the size and position of metal tip reasonably, the discharge can be generated in the combustor.
(4) During the ignition via discharge, due to the high electron density, the plasma has a greater influence on the phase of microwave propagation. With the increase in volume of discharge kernel, the phase changing increases gradually, and the reflection coefficient thus increases. The electron density of flame is low, thus having little effect on the phase of microwave propagation; however, the reflection coefficient decreases with the increase in flame volume because the number of electrons increases with the increase in flame volume, which improves the absorption of microwave energy.
(5) Microwave can enhance the development of spark discharge, thus reducing the size of discharge kernel, which will lead to the increase in discharge power density and a wider ignition limit, as well as reducing the consumption of power for ignition. However, when the microwave energy is further increased, the stronger ionization will lead to the increase in size and the wrinkles of the discharge kernel, which can also improve the ignition and cause a turbulent combustion.

## Data Availability

The raw data supporting the conclusions of this article will be made available by the authors, without undue reservations.
